# Supplementary material for: Emergence of winner-takes-all connectivity paths in random nanowire networks
Source: Nat Commun. 2018 Aug 13;9:3219. doi: 10.1038/s41467-018-05517-6 (PMC6089893; doi:10.1038/s41467-018-05517-6)
Supplement: Supplementary file 1 — Supplementary Information [file 41467_2018_5517_MOESM1_ESM.pdf]

Emergence of Winner-takes-all Connectivity Paths in Random Nanowire Networks

H. G. Manning et al.

Supplementary Information

Supplementary Figures

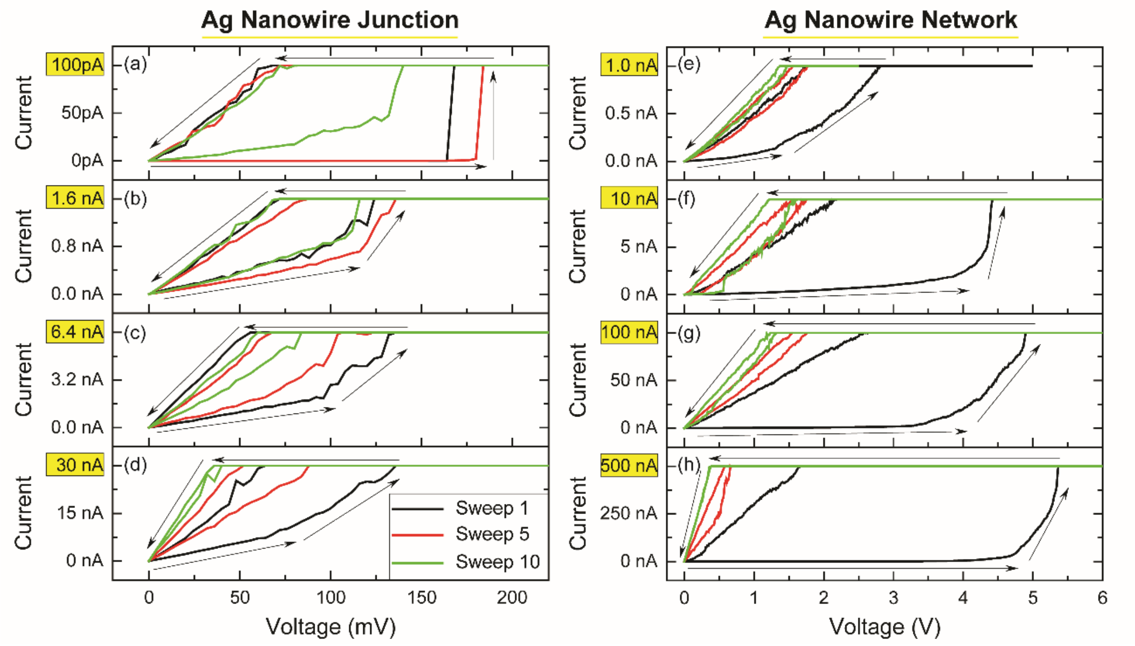

Supplementary Figure 1: (Color online) Electrical characterization of nanowire (NW) systems. The panels show experimentally obtained I-V curves for 10 successive sweeps (sweep 1,5 and 10 shown in black, red and green, respectively) for a single Ag nanowire junction (a-d) and a 500 µm Ag nanowire network (NWN) (e-h) at increasing current compliance levels. Large hysteresis loops are observed for low current compliance levels (a-b) with hysteresis loops collapsing after an increasing number of sweeps when larger currents are passed through the device (c-d). The collapse of the hysteretic loops is more pronounced in the Ag NWNs (e-h). After the initial sweep (black curve) a much higher leakage current is collected for the subsequent measurements. In all cases, between each set of 10 sweeps, the system was allowed to relax for a short period of time (~ 1 minute) which causes the hysteresis loops to reopen.

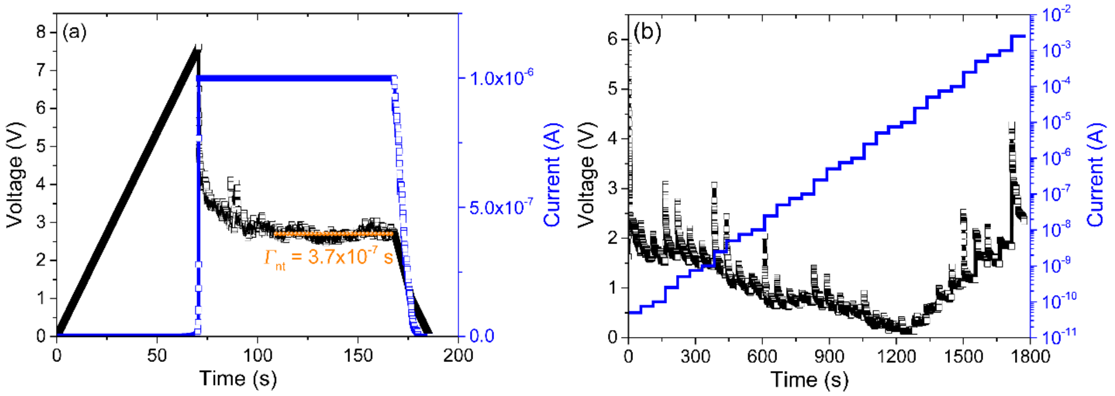

Supplementary Figure 2: Measured voltage and current plots for a 200 x 200 µm Ag nanowire network (NWN). (a) An increasing voltage is sourced across the Ag NWN as shown by the increasing black line, as a conducting pathway is formed through the network and current flows; the device quickly reaches a preset current compliance (1 µA, shown in blue) at 7.7 V. The subsequent drop in the measured voltage is required to maintain the 1 µA compliance level. The conductance of the network, $\mathit{\Gamma}_{\mathrm{nt}}$, is taken as an average over the region before the current returns from the compliance limit, shown by the orange dashed line. This measurement was repeated at increasing current compliance levels resulting in the conductance vs current compliance plots shown in Figure 1(e) of the main text. (b) Conductance plateaus found in Supplementary Figures 4 (a-b) were obtained using a current step measurement, where a constant current is sourced in a stepwise manner (shown in blue) with the voltage required to maintain this current captured by the black data points. $\mathit{\Gamma}_{\mathrm{nt}}$ was obtained for each compliance value as an average after the initial spike and decay of the applied voltage. The minimum applied voltage at approximately 1200 s represents the winner-takes-all (WTA) path and plateau region where the network dissipates the lowest power possible. The subsequent increase in voltage is required to form additional pathways.

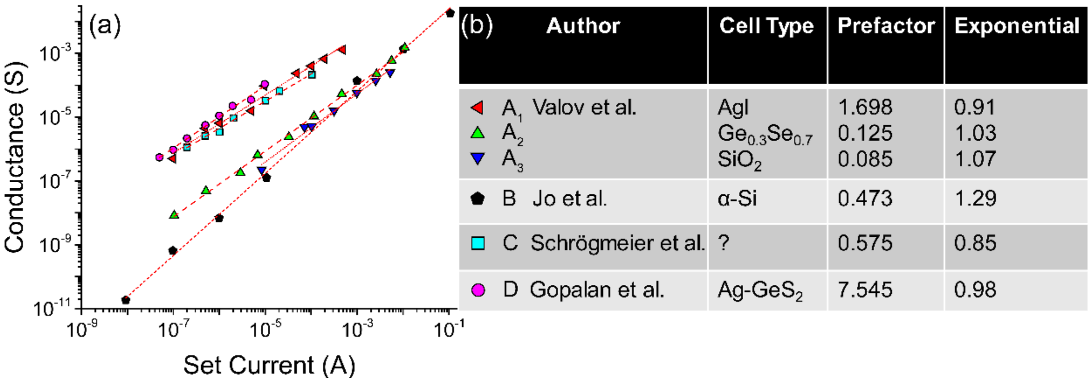

(b)	Author	Cell Type	Prefactor	Exponential
◀ A_1	Valov et al.	AgI	1.698	0.91
▲ A_2		$Ge_{0.3}Se_{0.7}$	0.125	1.03
▼ A_3		SiO_2	0.085	1.07
⬟ B	Jo et al.	α-Si	0.473	1.29
□ C	Schrögmeier et al.	?	0.575	0.85
● D	Gopalan et al.	Ag-GeS_2	7.545	0.98

Supplementary Figure 3: Conductive-bridge random-access-memory conductance versus set current scaling of thin film cells found in the literature. (a) Differing materials show similar exponents all around one. (b) Table of material systems containing prefactors and exponents taken from Valov et al. [1], Jo et al. [2], Schrögmeier et al. [3], and Gopalan et al. [4], as depicted in (a).

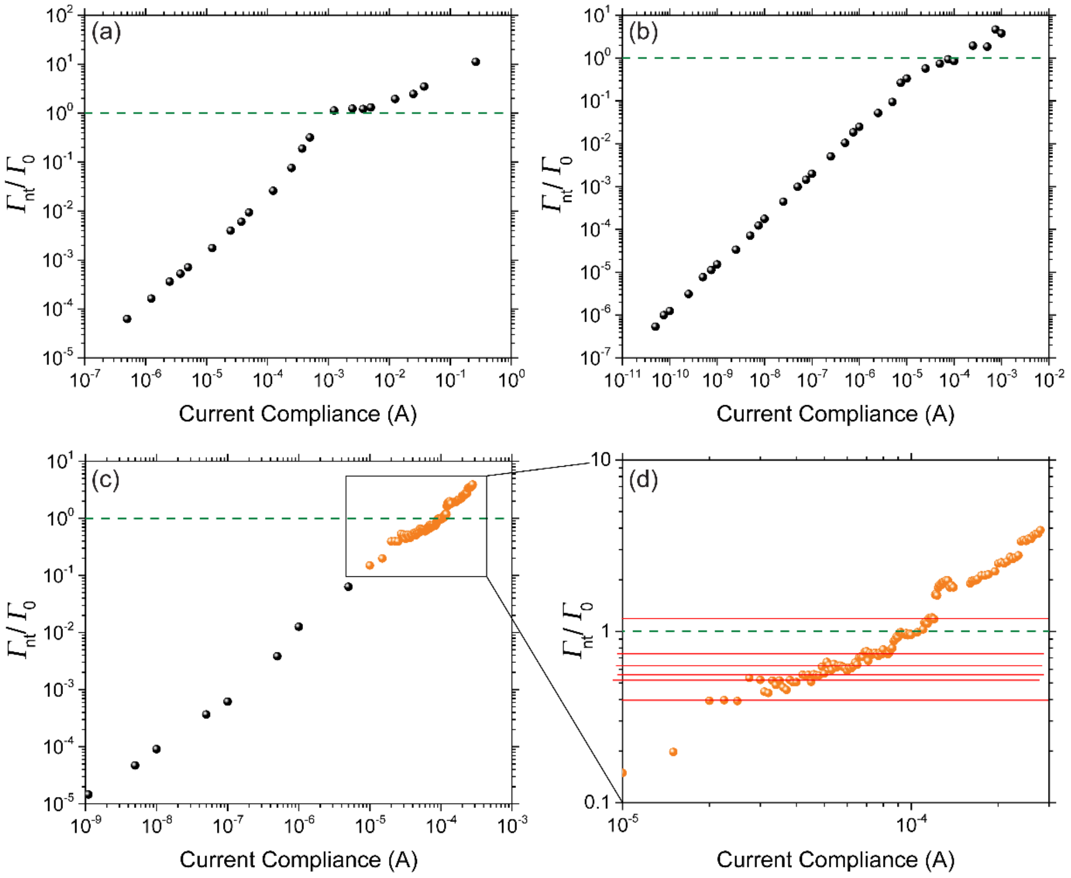

Supplementary Figure 4: Experimentally observed plateaus in Ag and Cu nanowire networks (NWNs). The panels show the ratio $\mathit{\Gamma}_{\mathrm{nt}}/\mathit{\Gamma}_0$ where $\mathit{\Gamma}_{\mathrm{nt}}$ is the conductance of the NWN and $\mathit{\Gamma}_0$ is the quantum of conductance plotted against current compliance (log-log scale) with conductance plateaus for Ag NWNs of size (a) 500 x 500 µm and (b) 100 x 100 µm. (c) $\mathit{\Gamma}_{\mathrm{nt}}/\mathit{\Gamma}_0$ versus current compliance values from a 20 x 20 µm Cu NWN. (d) Magnification of the highlighted data points in panel (c) showing the fine current compliance sampling which detects the numerous conductance plateaus marked by the horizontal red lines. The dashed line marks $\mathit{\Gamma}_{\mathrm{nt}}/\mathit{\Gamma}_0 = 1$.

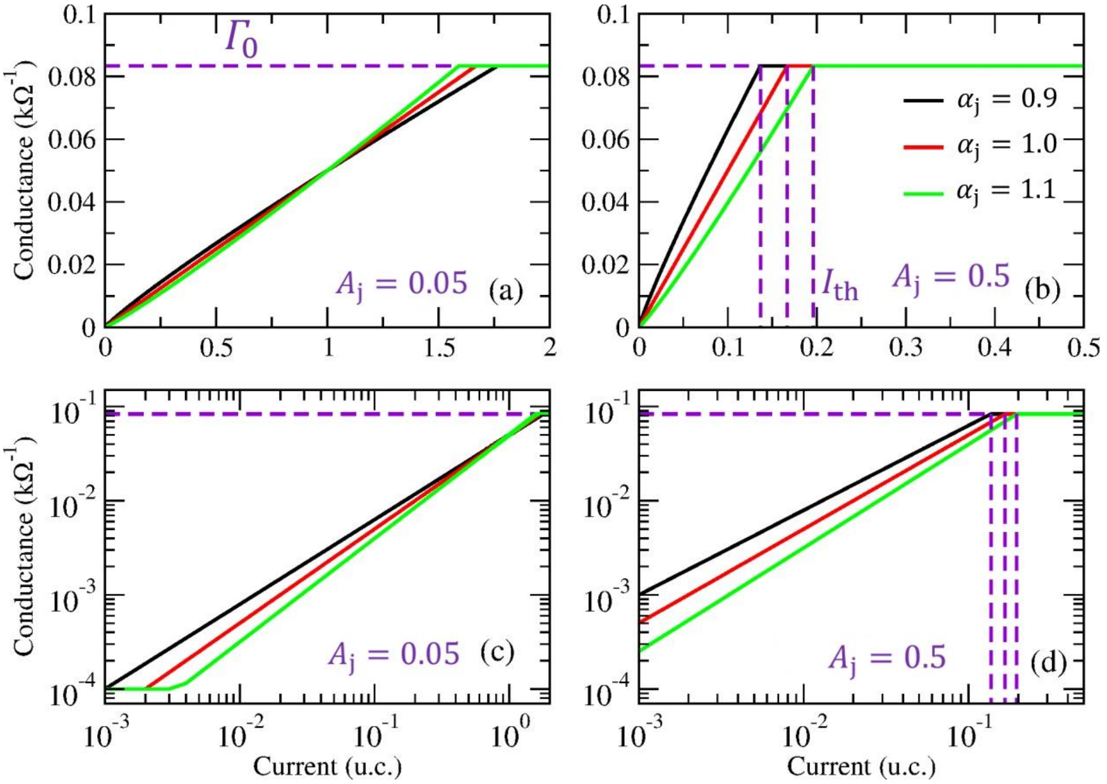

Supplementary Figure 5: (Color online) Graphic representation of the power-law plus cut-offs (PL+C) model for individual junctions. Conductance versus current plots taken for a single junction at linear (a-b) and logarithmic (c-d) scales. The current is expressed in units of current (u.c.). The curves were taken using PL+C model as specified in the main text. The prefactor value used in the panels (a) and (c) was $A_\mathrm{j} = 0.05$ and in the panels (b) and (d) was $A_\mathrm{j} = 0.5$. Horizontal dashed lines mark the quantum of conductance (Γ_0) whereas vertical ones mark the current thresholds up to where a junction displays memristive features. This is given by $I_\mathrm{th} = \left(1/A_\mathrm{j}R_\mathrm{on}\right)^{1/\alpha_\mathrm{j}}$.

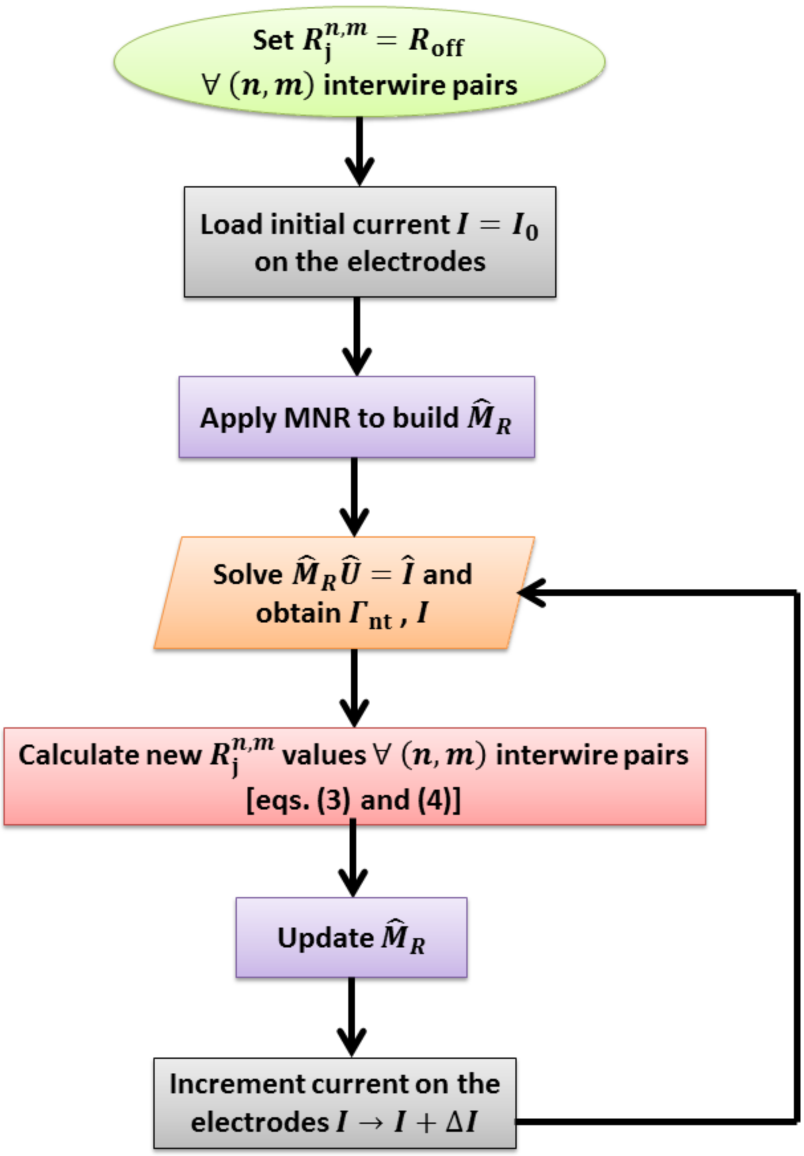

Supplementary Figure 6: "Part-to-whole" computational algorithm for nanowire networks (NWNs) and network templates. Schematics of the computational implementation of power-law plus cut-offs (PL+C) junction model onto macroscopic networks. Equations (3) and (4) specified in this workflow refers to equations (3) and (4) appearing in the main text. The algorithm obtains the conductance evolution of NWNs subjected to an electrical current source. See main text for detailed explanation of the algorithm.

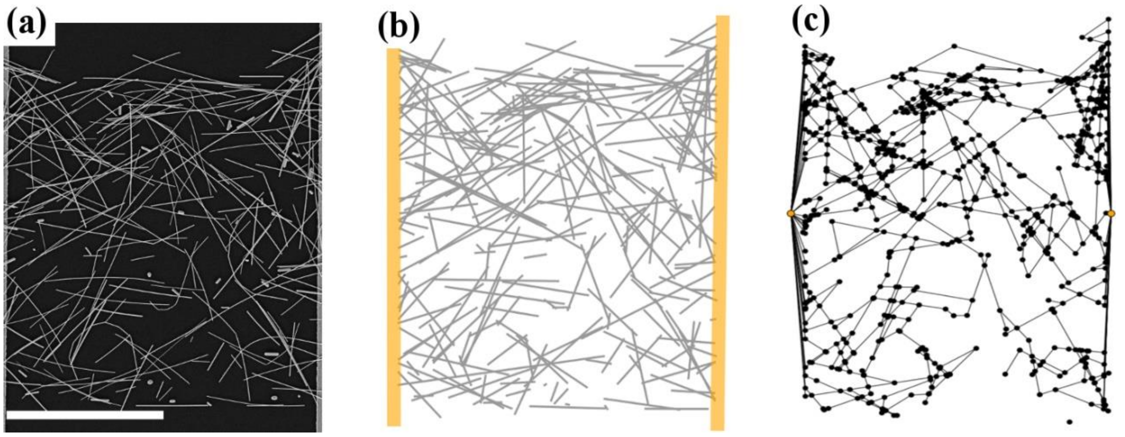

Supplementary Figure 7: Digitisation of experimental to computational network. (a) Scanning electron micrograph (SEM) of a silver nanowire network (NWN) containing 0.49 nanowires/μm^2. Bottom scale bar represents 10 μm. (b) Computational transcription of the network after image processing the image in (a). Wires are represented by grey sticks and electrodes are represented by vertical orange bars. (c) Graph representation of the virtual NWN that serves as a template for the current maps shown in Figures 3 (main text), Supplementary Figure 9, and Supplementary Figure 10.

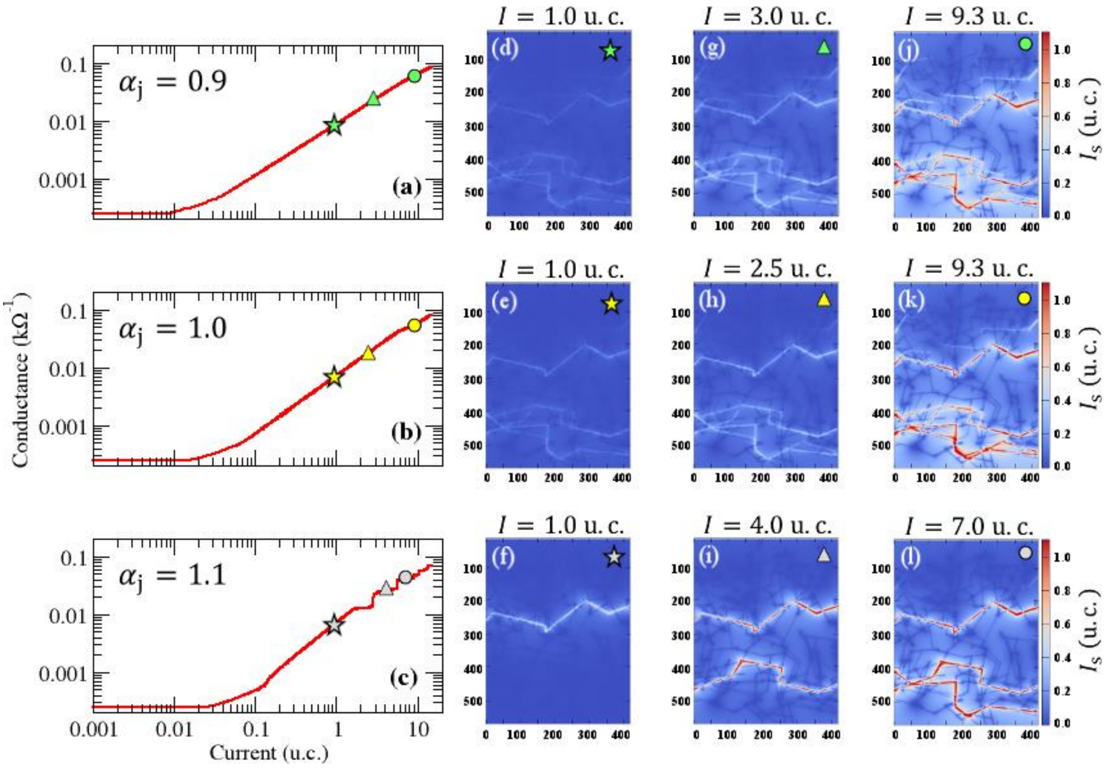

Supplementary Figure 8: Conductance versus current curves for an Ag nanowire network (NWN) sample containing 0.47 nanowires/μm^2. The junctions were described with $A_j = 0.05$ and exponents (a) $\alpha_j = 0.9$, (b) $\alpha_j = 1.0$, (c) $\alpha_j = 1.1$. The symbols mark points in the curves in which current colour maps (panels d-l) were taken. (d-l) Current colour maps calculated over each wire segment (I_s) of the Ag NWN. Snapshots were taken for three current values with units of current (u.c.) specified on the top of each current map and distinguished by the symbols: star (set in the power-law regime), and triangle and circle (both set in the post power-law regime). Animations revealing the complete evolution of the network in response to the current source, junction optimization of the top-3 paths of least-resistance, and current-segment maps are provided in the supplemental material (cf. Supplementary Note 3 of this document for animation description).

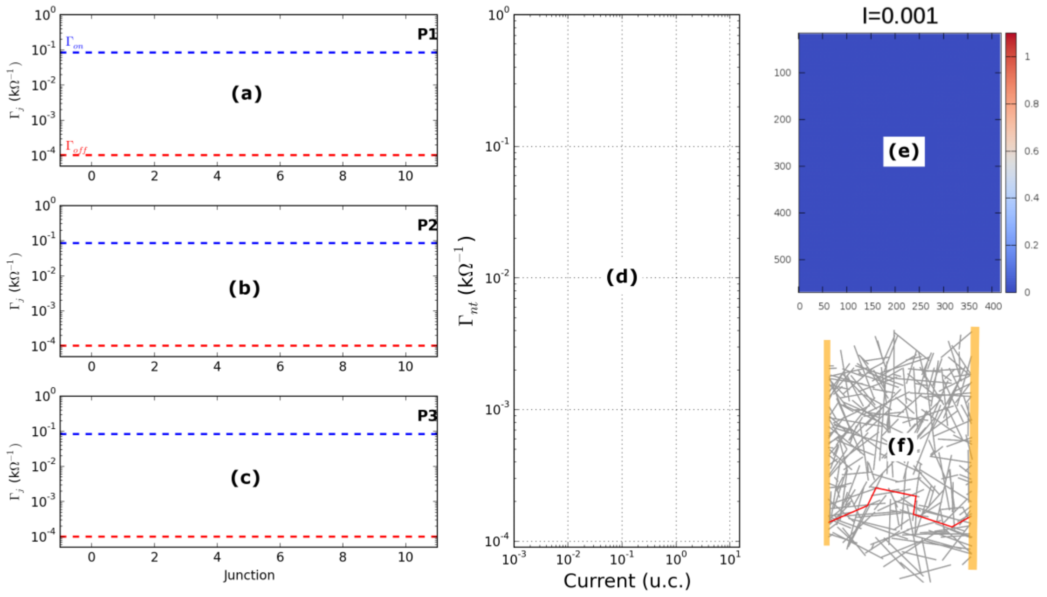

Supplementary Figure 9: Snapshot template of the animation revealing the conductance evolution of a nanowire network (NWN) as a function of current. Panels (a-c) monitor the conductance of the junctions (Γ_{j}) that compose the top-3 paths carrying most of the current load. Junctions are labelled as integer numbers in the abscissa. The conductance values are displayed as level-bars that are free to move in the range of $[\Gamma_{\mathrm{off}}, \Gamma_{\mathrm{on}}]$ marked by horizontal dashed lines. Panel (d) shows the whole Γ_{nt} versus I curve with a dynamical marker (red circle) that tracks the evolution of the simulation. (e) Current-segment maps as those depicted in Figures 3 (main text), Supplementary Figure 8, Supplementary Figure 9, and Supplementary Figure 10. (f) Digitalized image of the NWN with the top-3 paths being highlighted with red sticks. A description of the animation can be found in the Supplementary Note 3.

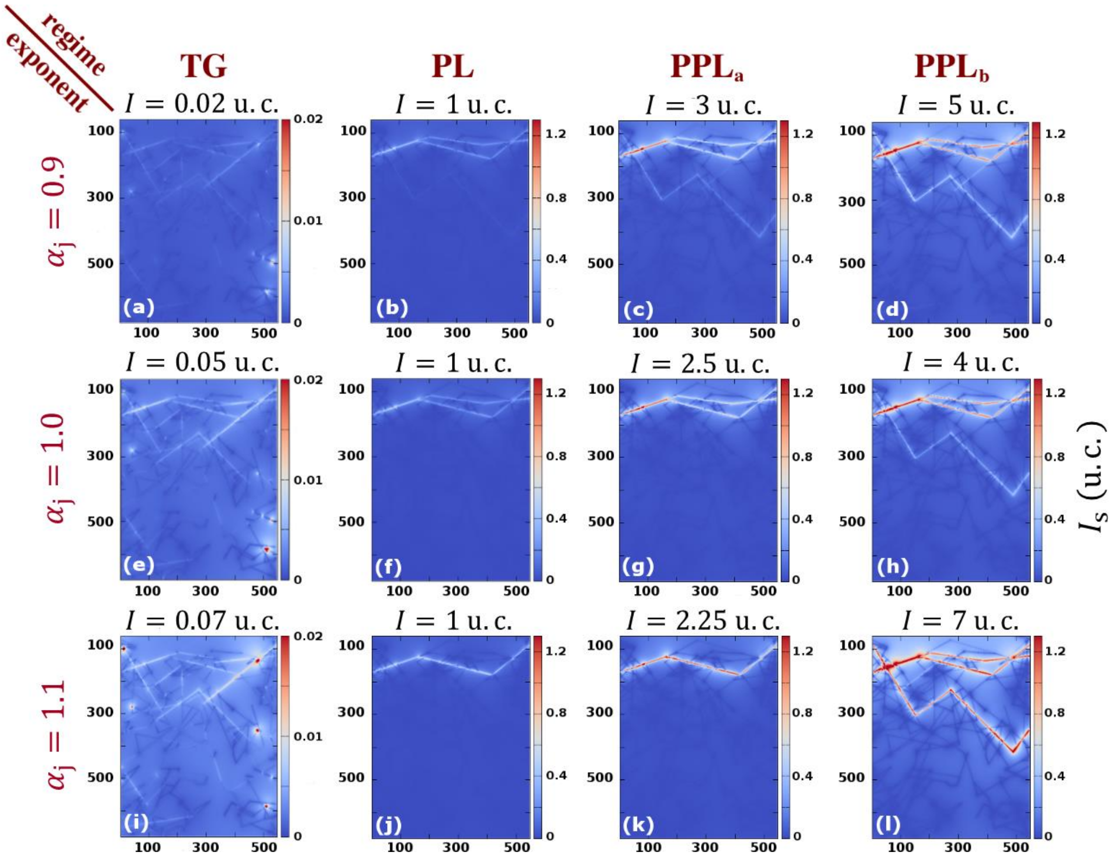

Supplementary Figure 10: Current colour maps calculated over each wire segment (I_s) of the Ag nanowire network (NWN) in Supplementary Figure 7. Snapshots are arranged in a grid in which its abscissa refers to the conducting regime and its ordinate refers to the junction exponent. These maps were obtained for a fixed prefactor value of $A_\mathrm{j} = 0.05$ and for distinct junction exponents: (a-d) $\alpha_\mathrm{j} = 0.9$, (e-h) $\alpha_\mathrm{j} = 1.0$, (i-l) $\alpha_\mathrm{j} = 1.1$. The units of current (u.c.) values are specified on each panel for which the snapshots were taken in the transient growth (TG) phase, power-law (PL), and two post-power-law (PPL, labelled with subscript a and b) regimes. Panels (i-l) correspond to the same results shown in Figure 3 in the main text. Animations revealing the complete evolution of the network in response to the current source, junction optimization of the top-3 paths of least-resistance, and current-segment maps are provided in the supplemental material (cf. Supplementary Note 3 of this document for animation description).

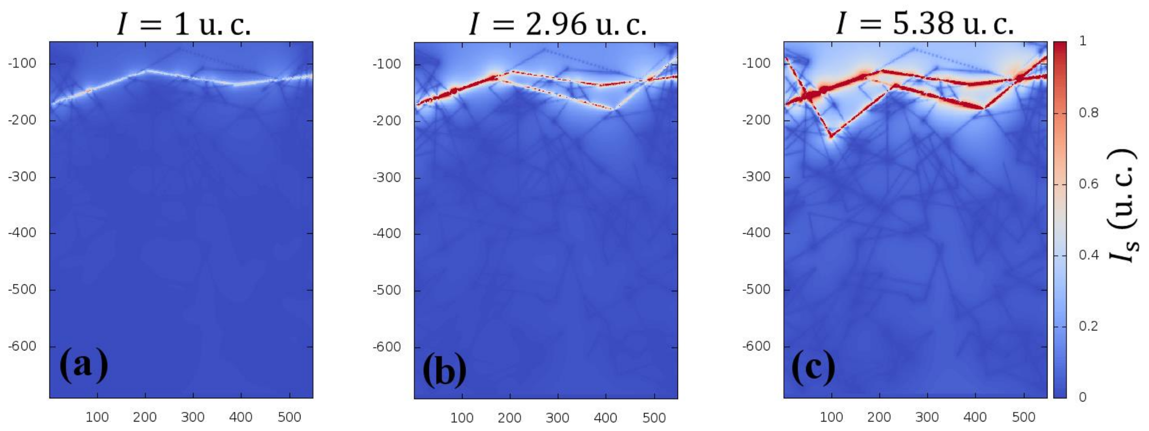

Supplementary Figure 11: Current maps for a nanowire network (NWN) with disorder in the junction exponents and quick activation element. Current colour maps calculated over each wire segment (I_s) of the Ag NWN depicted in the Supplementary Figure 7. The junction settings are for a heterogeneous NWN with $A_j = 0.05$ and a narrow dispersion was induced in the α_j exponents using a normal distribution with $\langle\alpha_j\rangle = 1.05$, $\sigma = 0.1$ and truncated at $[1.0,1.1]$. Snapshots were taken for three input currents (expressed in units of current, u.c.) corresponding to different conducting regimes: (a) $I = 1$ u. c. at the power-law regime, and (b) $I = 2.96$ u. c. and (c) $I = 5.38$ u. c., both at the post-power-law regime.

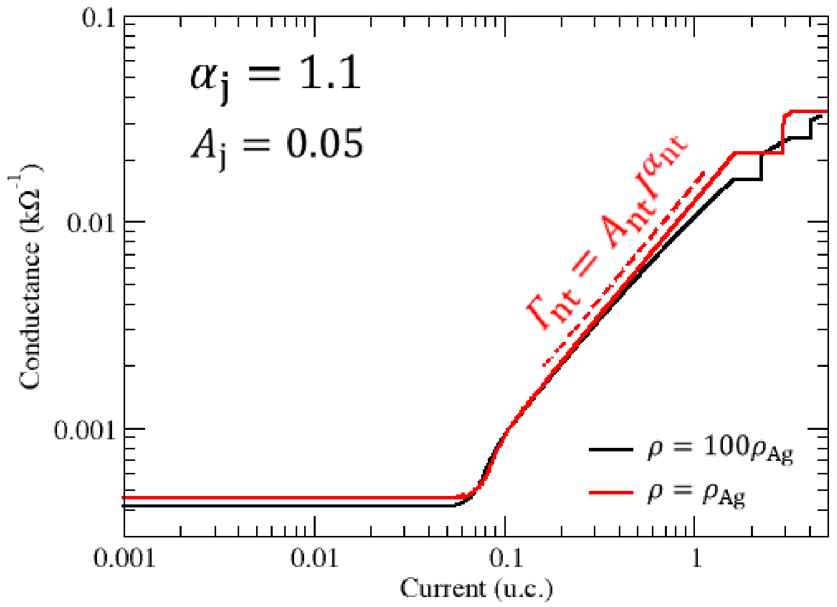

Supplementary Figure 12: Conductance versus current curves taken for the Ag nanowire network (NWN) shown in Supplementary Figure 7 with wire resistivity of $\rho = \rho_{Ag} = 22.6 \pm 2.3$ nΩm (red line) and $\rho = 100\rho_{Ag}$ (black line). The dashed red line shows a power law fitting of $\Gamma_{nt} = A_{nt}I^{\alpha_{nt}}$ with $A_{nt} = 0.0125$ and $\alpha_{nt} = 1.1$. Currents are expressed in units of current (u.c.). The junctions were described using $A_j = 0.05$ and $\alpha_j = 1.1$.

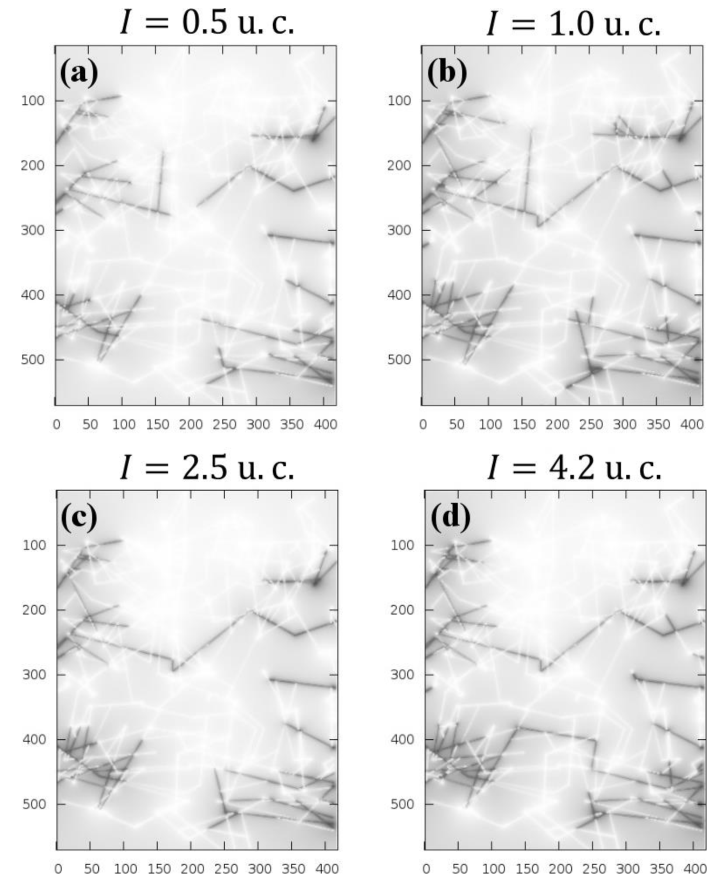

Supplementary Figure 13: Binary activation maps taken for the Ag nanowire network (NWN) investigated in Supplementary Figure 8. Four snapshots were obtained for distinct input current values written in units of current (u.c.): (a) $I = 0.5\ u.c.$, (b) $I = 1.0\ u.c.$, (c) $I = 2.5\ u.c.$, and (d) $I = 4.2\ u.c.$. Only wires carrying an amount of current above a certain threshold value (0.009 u.c.) were contrasted in dark colour. This result was taken with the assumption that nanowires in the vicinity of the source electrode activate faster than the ones located in the mid-section of the network. Note the more imperfect profile of the winner-takes-all (WTA) state - which emerges at $I = 2.5\ u.c.$ - with numerous wires branching out of the electrodes as a result of current leakage to other parts of the network. Yet, this WTA state manages to carry about 81% of the total current as quantified within our simulations.

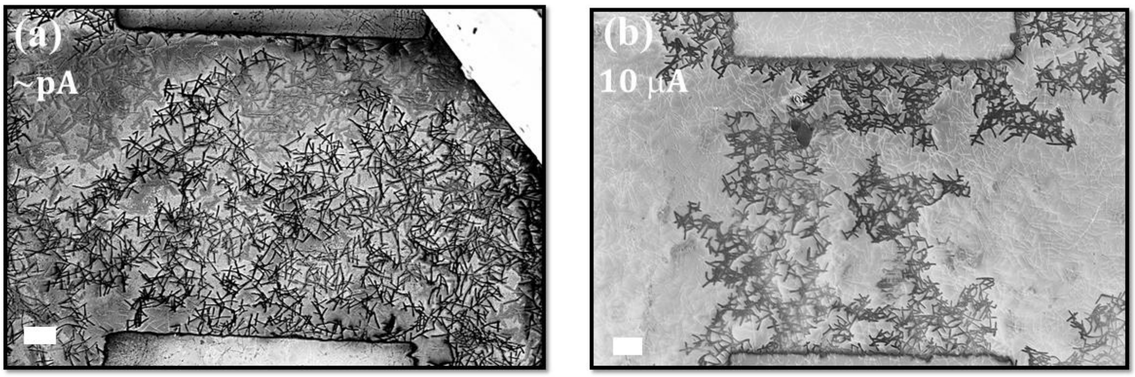

Supplementary Figure 14: Passive voltage contrast (PVC) scanning-electron microscope (SEM) images of two distinct Ag nanowire network samples of dimensions 200 x 200 μm. In (a), the image was taken by holding the source voltage of 2 V resulting in a leakage current of a few hundreds of pA (see label on image). In panel (b), the image was taken during an I-V sweep (electrodes grounded) with limiting current compliances of 10 μA. Current levels are written on the respective panels. White scale bars correspond to 20 μm. Note a PVC image provides a qualitative comparison of the electrical connectivity within a given network. It is not possible to compare the contrast observed in different networks or even that observed in the same network imaged under different conditions.

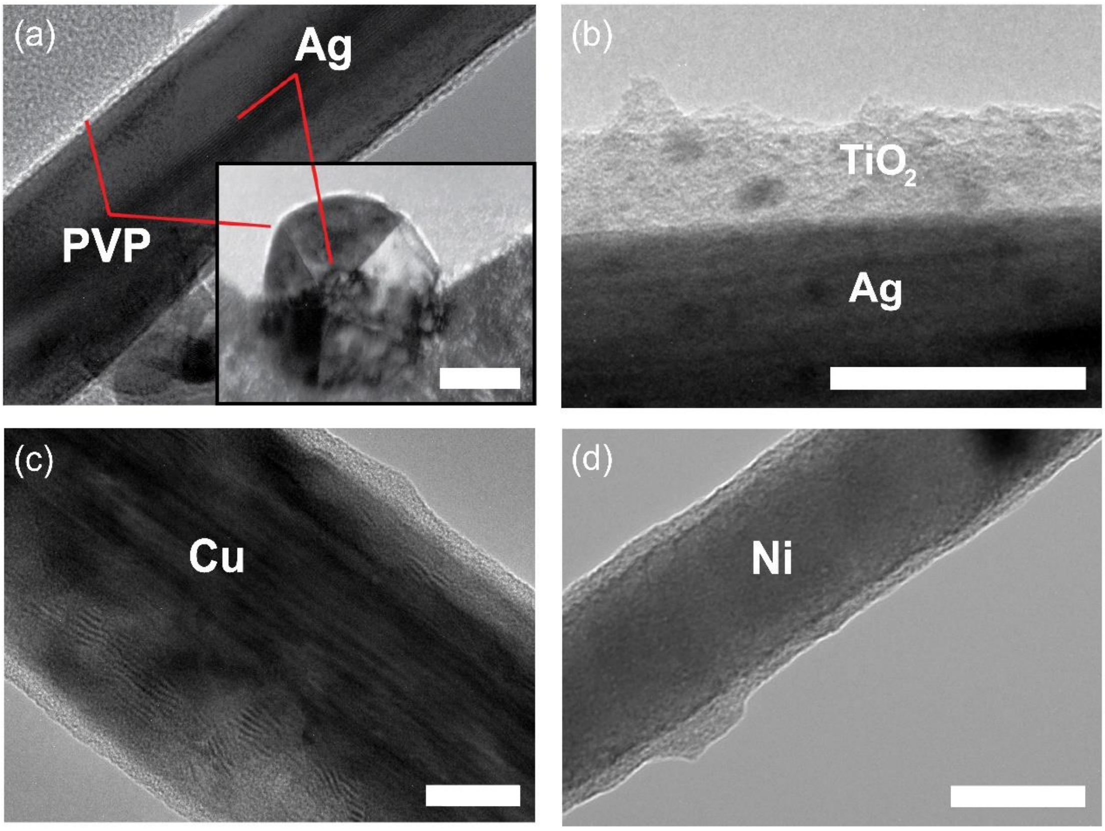

Supplementary Figure 15: Transmission electron microscopy of metallic core-shell nanowires described in Supplementary Note 1. (a) Ag nanowires with a Polyvinylpyrrolidone (PVP) coating. The inset shows the pentagonally twinned structure of the nanowire cross-section. The scale bar represents 20 nm. (b) Ag core TiO_2 shell (Ag@TiO_2) nanowire. The dark inclusions in the TiO_2 shell are regions of Ag in the amorphous TiO_2 shell. The scale bar corresponds to 100 nm. (c) Cu and (d) Ni nanowires both form a thin native oxide shell. The scale bars represent 20 nm and 50 nm for panels (c) and (d), respectively.

Supplementary Tables

Supplementary Table 1: Average exponent (α) and prefactor (A) values obtained experimentally for a range of single nanowires, nanowire junctions (Jxn) and nanowire networks (NWN). All values were taken over ensembles of material samples except for the TiO_2 single wire from which only one sample was experimented.

System Type	Material	α	A
**NWN (200 x 200 μm)**	Ag	1.10 ± 0.07	9.56 ± 10.24
**Jxn**	Ag	1.05 ± 0.05	32.29 ± 15.58
**NWN (20 x 20 μm)**	Cu	0.97 ± 0.03	0.49 ± 0.43
**Jxn**	Cu	1.05 ± 0.07	3.55 ± 2.92
**Jxn**	$AgTiO_2$	1.03 ± 0.02	7.69 ± 4.77
**NWN (50 x 50 μm)**	Ni	1.01 ± 0.03	0.20 ± 0.21
**Single wire**	Ni	0.95 ± 0.01	0.29 ± 0.03
**Single wire**	TiO_2	0.83	0.003

Supplementary Notes

Supplementary Note 1:

Summary of Nanowires Systems Studied in this Work

Ag Nanowires

Single crystalline pentagonal twinned Ag nanowires (NWs) (Seashell Technology) with a thin polymeric Polyvinylpyrrolidone (PVP) coating from synthesis were used in all experiments. Transmission electron microscopy (TEM) image shown in Supplementary Figure 15(a) confirms the crystal structure of the NW and the PVP coating (1-2 nm thick).

Ag-TiO_2 Nanowires

Ag-TiO_2 NWs were synthesized using a solvothermal growth method as previously reported [5]. These wires have an Ag core with an amorphous TiO_2 coating. Supplementary Figure 15(b) displays TEM images of the Ag core and the amorphous crystallinity of the TiO_2 shell.

Cu Nanowires

Cu NWs were synthesized using the method described by Rathmell et al. [6] Cu NWs develop a thin oxide layer through NW processing and exposure to ambient conditions. TEM analysis shown in Supplementary Figure 15(c) of the NWs shows the Cu metal core with oxide coating (~ 5.3 nm).

Ni Nanowires

Ni NWs (Nanomaterials.it) consisted of a metal core with a native amorphous oxide coating of around 4-8 nm in thickness.

TiO_2 Nanowires

TiO_2 NWs (Nanomaterials.it) consisted of a metal core with a native amorphous oxide coating of around 4-8 nm in thickness.

Collection of exponents and prefactors obtained experimentally

The power law scaling behaviour $\Gamma = A\, I_c^{\alpha}$ is ubiquitous across a wide range of nanoscale junction arrangements. Supplementary Table 1 shows data for the scaling exponent α and the prefactor A for single wires, single junctions and networks made of Ag, Cu, Ni, TiO_2 and core shell Ag-TiO_2 nanowires. Physical representations of α and A are discussed in the Supplementary Note 2.

Supplementary Note 2:

Relationship with the ion-drift model

A popular description of a single memristive system was elaborated by Strukov et al. [7] in which an ion drift mechanism was used to verify the memristor fingerprints of dynamical TiO_x-based junctions. Changes in the resistance of the junction are attributed to an effective modulation of the interfacial barrier splitting a doped (TiO_{2-x}) and an undoped (TiO_2) layer upon application of an electric field. This modulation is caused by the drift of oxygen vacancies across the interface in response to the applied electric field. Following the linear ionic drift in a uniform field assumption, the electrical response of the junction is given by

$$V(t) = \left[R_{\mathrm{on}}\frac{w(t)}{D} + R_{\mathrm{off}}\left(1 - \frac{w(t)}{D}\right)\right] I(t) \qquad (1)$$

$$\frac{dw}{dt} = \mu_{\mathrm{v}}\frac{R_{\mathrm{on}}}{D} I(t) \qquad (2)$$

where t is the time, D is the full length of the junction, μ_{v} is the ion mobility, I is the input current, and V is the output potential (considering that the system is current-driven). w is a state variable representing the varying length of the doped layer.

We will now demonstrate how the state equation (Supplementary Equation 2) can be obtained from the power-law relation in equation (1) (main text) considering another proxy for the conductance scaling. According to our power-law plus cut-offs (PL+C) description, the conductance of a single junction is a dynamical quantity whose value is regulated by the current compliance. For each I_{c} value, a current versus time curve is unfolded out of a full voltage sweep; its area is given by $Q_{\mathrm{c}} = \int_{-\infty}^{t} I(\tau)d\tau$ where Q_{c} is the total amount of charge flowing through the junction during the time period of a full voltage sweep given by t. Therefore, an increment in I_{c} yields an increment in Q_{c} in such a way that $Q_{\mathrm{c}} \propto I_{\mathrm{c}}$. Without loss of generality, equation (1) (main text) can be then written in terms of the cumulative charge Q_{c} such as,

$$\Gamma_{\mathrm{j}} = A_{\mathrm{j}} Q_{\mathrm{c}}^{\alpha_{\mathrm{j}}} \qquad (3)$$

with the Q_{c} being the new proxy variable of the power-law (PL). In fact, all our experimental results taken for junctions and networks were minimally affected with respect to plotting the conductance data as a function of Q_{c} or I_{c}.

The junctions studied in this work spend most of their lifespan "stabilized" in the non-resonant tunnelling regime in which their conductance follows an exponential dependency with the electrode separation,

$$\Gamma_{\mathrm{j}}(t) = \Gamma_0 e^{-\beta[D-w(t)]} \qquad (4)$$

where β is the decay parameter that characterizes the tunnelling barrier and $w(t)$ here represents the length of the conducting filament. For sufficiently small tunnelling separations and considering the simplest case in which $\alpha_{\mathrm{j}} = 1$, we can approximate Supplementary Equation 4 up to first order and related it with Supplementary Equation 3 such as

$$\Gamma_{\mathrm{j}}(t) \approx \Gamma_0\left[1 - \beta\left(D - w(t)\right)\right] = A_{\mathrm{j}} Q_{\mathrm{c}}(t) \qquad (5)$$

Performing the derivative in time of Supplementary Equation 5 we obtain

$$\frac{d\Gamma_{\mathrm{j}}}{dt} = \Gamma_0 \beta \frac{dw}{dt} = A_{\mathrm{j}} \frac{dQ_{\mathrm{c}}}{dt} \qquad (6)$$

which gives the following state equation ruling the filament growth

$$\frac{dw}{dt} = \frac{A_{\mathrm{j}}}{\beta \Gamma_0} I(t) \qquad (7).$$

Considering that $R_{\mathrm{on}} = 1/\Gamma_0$, the comparison between Supplementary Equation 2 and Supplementary Equation 7 provides

$$A_{\mathrm{j}} = \frac{\mu_{\mathrm{v}} \beta}{D} \qquad (8).$$

The effects of nonlinearity in the charge carrier drift can be captured by repeating the whole derivation above for $\alpha_{\mathrm{j}} \neq 1$. Using Supplementary Equation 3 and Supplementary Equation 4, we get the generalized state equation

$$\frac{dw}{dt} = \frac{\mu_{\mathrm{v}}}{D\Gamma_0} I(t)\, e^{\beta[D-w(t)]}\, \alpha_{\mathrm{j}} [Q_{\mathrm{c}}(t)]^{\alpha_{\mathrm{j}}-1} = \frac{\mu_{\mathrm{v}}}{D\Gamma_0} I(t)\, f\left(w/D\,, \alpha_{\mathrm{j}}, Q_{\mathrm{c}}(t)\right) \qquad (9).$$

Note that the first order approximation in Supplementary Equation 4 was not taken in this derivation rendering hence the exponential term in expression Supplementary Equation 9. This result is analogue to other generalizations of the ion-drift model [7, 8, 9, 10, 11, 12] in which window functions are used to account for the nonlinear effects existent at the boundaries of the conducting channel and for the nonlinear dependency of the state derivative on the driven current.

Supplementary Note 3:

Description of the animation supporting files

Animations revealing the complete evolution of the network in response to the current source are provided in numerous supporting files. Each snapshot contains six panels as explained in the sketch of Supplementary Figure 9.

The names of the animation files and their respective initial conditions are described below:

- Supplementary Movie 1: animation for the Ag nanowire network (NWN) shown in Supplementary Figure 7 with $A_{\mathrm{j}} = 0.05$ and $\alpha_{\mathrm{j}} = 1$. One can see the formation of two superimposed conductive paths in the first power-law regime. Subsequent paths are formed as current increases causing the observed changes in slopes in the conductance curve.
- Supplementary Movie 2: animation for the Ag NWN shown in Supplementary Figure 7 with $A_{\mathrm{j}} = 0.05$ and $\alpha_{\mathrm{j}} = 1.1$. One can see the formation of a single conductive path in the power-law regime. Once all the junctions in this path are fully optimized, the network becomes temporarily Ohmic, i.e. its conductance does not change within a certain current window. Further paths are formed in a quantized manner as current is loaded onto the electrodes with the conductance curve depicting a stepwise increase.
- Supplementary Movie 3: animation for the Ag NWN used in Supplementary Figure 8 with $A_{\mathrm{j}} = 0.05$ and $\alpha_{\mathrm{j}} = 1$. One can see the formation of multiple conductive paths in the first power-law regime. Subsequent paths are formed as current increases causing the observed changes in slopes in the conductance curve.
- Supplementary Movie 4: animation for the Ag NWN used in Supplementary Figure 8 with $A_{\mathrm{j}} = 0.05$ and $\alpha_{\mathrm{j}} = 1.1$. One can see the formation of a single conductive path practically slicing the network at half in the power-law regime. Once all the junctions in this path are fully optimized, the network becomes temporarily Ohmic, i.e. its conductance does not change within a certain current window. After the first conductance plateau, one can observe the formation of two independent conductive paths. As more current is loaded onto the terminals, additional paths are formed in a quantized manner with the conductance curve depicting a stepwise increase.

Supplementary Note 4:

Influence of NWN inner resistance on the self-similar behaviour

Let's assume the simplified case in which the network selects M identical parallel paths, each path containing the same number of wires N. Therefore, each path contains N segments and $N+1$ junctions (including the two extra junctions that the wire path makes with the electrodes). Let's also consider that all wire segments have nearly the same length. The resistance of a single path (R_p) can be then written as

$$R_\mathrm{p} = NR_\mathrm{in} + (N+1)R_\mathrm{j} \qquad (10).$$

Substituting $R_\mathrm{j} = i^{-\alpha_\mathrm{j}}/A_\mathrm{j}$ (junctions ruled by a power law) into Supplementary Equation 10, we obtain

$$R_\mathrm{p} = NR_\mathrm{in} + (N+1)\frac{i^{-\alpha_\mathrm{j}}}{A_\mathrm{j}} \qquad (11)$$

being i the amount of current flowing through the path, i.e. $i = I/M$ with I being the current sourced in the electrodes. We can then write down the equivalent conductance for M parallel paths as

$$\Gamma_\mathrm{eq} = \Gamma_\mathrm{nt} = \frac{M}{R_\mathrm{p}} \qquad (12).$$

Further manipulation of Supplementary Equation 12 gives

$$\Gamma_\mathrm{nt} = \left(\frac{MA_\mathrm{j}}{N+1}\right) i^{\alpha_\mathrm{j}} \left[\frac{1}{1+\left(\frac{NR_\mathrm{in}A_\mathrm{j}}{N+1}\right) i^{\alpha_\mathrm{j}}}\right] \qquad (13)$$

which shows that the dominant contribution for the network conductance in the limit of low inner wire resistances ($R_\mathrm{in} \ll R_\mathrm{j}$) is a power-law with $\alpha_\mathrm{nt} = \alpha_\mathrm{j}$ and $A_\mathrm{nt} = M^{1-\alpha_\mathrm{j}}A_\mathrm{j}/(N+1)$. The term inside the square brackets in Supplementary Equation 13 will play a relevant role when the skeleton of the network becomes more robust, i.e. R_in increases. In this case one expects a breakdown in the self-similar behaviour as the functional form of $\Gamma_\mathrm{nt}(I)$ does not correspond to a pure power law. To confirm this point, we conduct extra calculations on the same network of Supplementary Figure 7 except that the resistivity of the wires was artificially increased as $\rho = 100\rho_\mathrm{Ag}$. Supplementary Figure 12 shows the comparison between the real ($\rho = \rho_\mathrm{Ag}$) and the hypothetical network ($\rho = 100\rho_\mathrm{Ag}$) with junctions described by $A_\mathrm{j} = 0.05$ and $\alpha_\mathrm{j} = 1.1$. One can see that the conductance of the network made with highly resistive wires shows a pronounced deviation from the pure power law $\Gamma_\mathrm{nt} \propto I^{1.1}$, confirming the premise

raised by Supplementary Equation 13.

Supplementary References:

[1] Valov I, Kozicki MN. Cation-based resistance change memory. *J. Phys. D* **46**, 074005 (2013).

[2] Jo SH, Lu W. CMOS compatible nanoscale nonvolatile resistance switching memory. *Nano Lett.* **8**, 392-397 (2008).

[3] Schrögmeier P, et al. Time discrete voltage sensing and iterative programming control for a $4F^2$ multilevel CBRAM. *2007 IEEE Symposium on VLSI Circuits Digest of Technical Papers*, DOI: 10.1109/VLSIC.2007.4342708.

[4] Gopalan C, et al. Demonstration of conductive bridging random access memory (CBRAM) in logic CMOS process. *Solid-State Electron.* **58**, 54-61 (2011).

[5] Manning HG, Biswas S, Holmes JD, Boland JJ. Nonpolar Resistive Switching in Ag@TiO_2 Core-Shell Nanowires. *ACS Appl. Mater. Interfaces* **9**, 38959-38966 (2017).

[6] Rathmell AR, Wiley BJ. The synthesis and coating of long, thin copper nanowires to make flexible, transparent conducting films on plastic substrates. *Adv. Mater.* **23**, 4798-4803 (2011).

[7] Strukov DB, Snider GS, Stewart DR, Williams RS. The missing memristor found. *Nature* **453**, 80-83 (2008).

[8] Wey T, Benderli S. Amplitude modulator circuit featuring TiO_2 memristor with linear dopant drift. *Electron. Lett.* **45**, 1103-1104 (2009).

[9] Joglekar YN, Wolf SJ. The elusive memristor: properties of basic electrical circuits. *Eur. J. Phys.* **30**, 661 (2009).

[10] Biolek Z, Biolek D, Biolkova V. SPICE model of memristor with nonlinear dopant drift. *Radioengineering* **18**, 210-214 (2009).

[11] Prodromakis T, Peh BP, Papavassiliou C, Toumazou C. A versatile memristor model with nonlinear dopant kinetics. *IEEE Trans. Electron* **58**, 3099-3105 (2011).

[12] Lehtonen E, Laiho M. CNN using memristors for neighborhood connections. *2010 12th International Workshop on Cellular Nanoscale Networks and their Applications (CNNA)*, DOI: 10.1109/CNNA.2010.5430304.
