# Supplementary material for: Emergence of winner-takes-all connectivity paths in random nanowire networks
Source: Nat Commun. 2018 Aug 13;9:3219. doi: 10.1038/s41467-018-05517-6 (PMC6089893; doi:10.1038/s41467-018-05517-6)
Supplement: Supplementary file 3 — Description of Additional Supplementary Files [file 41467_2018_5517_MOESM3_ESM.pdf]

Description of Additional Supplementary Files

File Name: Supplementary Movie 1
Description: Animation for the Ag nanowire network shown in Supplementary Figure 7 with A_j=0.05 and α_j=1. One can see the formation of two superimposed conductive paths in the first power-law regime. Subsequent paths are formed as current increases causing the observed changes in slopes in the conductance curve.

File Name: Supplementary Movie 2
Description: Animation for the Ag nanowire network shown in Supplementary Figure 7 with A_j=0.05 and α_j=1.1. One can see the formation of a single conductive path in the power-law regime. Once all the junctions in this path are fully optimized, the network becomes temporarily Ohmic, i.e. its conductance does not change within a certain current window. Further paths are formed in a quantized manner as current is loaded onto the electrodes with the conductance curve depicting a stepwise increase.

File Name: Supplementary Movie 3
Description: Animation for the Ag nanowire network used in Supplementary Figure 8 with A_j=0.05 and α_j=1. One can see the formation of multiple conductive paths in the first power-law regime. Subsequent paths are formed as current increases causing the observed changes in slopes in the conductance curve.

File Name: Supplementary Movie 4
Description: Animation for the Ag nanowire network used in Supplementary Figure 8 with A_j=0.05 and α_j=1.1. One can see the formation of a single conductive path practically slicing the network at half in the power-law regime. Once all the junctions in this path are fully optimized, the network becomes temporarily Ohmic, i.e. its conductance does not change within a certain current window. After the first conductance plateau, one can observe the formation of two independent conductive paths. As more current is loaded onto the terminals, additional paths are formed in a quantized manner with the conductance curve depicting a stepwise increase.
